# The Relationship among Government, Enterprise, and Public in Environmental Governance from the Perspective of Multi-Player Evolutionary Game

**DOI:** 10.3390/ijerph16183351

**Published:** 2019-09-11

**Authors:** Yingxin Chen, Jing Zhang, Pandu R. Tadikamalla, Xutong Gao

**Affiliations:** 1 School of Economics and Management, Harbin Engineering University, Harbin 150001, China; chenyxdingdang@hrbeu.edu.cn (Y.C.); 18686799296@163.com (X.G.); 2 School of Information Science and Engineering, University of Jinan, Jinan 250022, China; 3 Joseph M.Katz Graduate School of Business, University of Pittsburgh, PA 15260, USA; Pandu@katz.pitt.edu

**Keywords:** environmental governance, public participation, government supervision, multi-player evolutionary game, participation mechanism

## Abstract

Environmental governance is an important component of the national governance system. China’s current environmental problems are particularly complex. How to let the government, enterprises, and the public participate in environmental governance is the key to enhance the ability of environmental governance. Based on the evolutionary game theory, the interaction and influencing factors among enterprise pollution control, government supervision, and public participation are analyzed, and the empirical analysis is carried out based on China’s 30 provincial panel data from 2009 to 2018. The research results show that government supervision has a positive effect on the environmental governance and can urge enterprises to actively perform pollution control. The effect of government supervision is constrained by the income and cost of enterprises, and the penalties for passive pollution control should be raised. At the same time, improving the government’s reputation loss can effectively stimulate the government’s environmental supervision behavior. Public participation significantly promotes the governance effect of three industrial wastes, and the enthusiasm of public participation is closely related to participation cost and psychological benefits. Public participation can replace government supervision to a certain extent. The interaction between government and public has a positive effect on environmental governance. The research results will help to build an effective environmental governance system and improve environmental governance performance and public satisfaction.

## 1. Introduction

With the development of China’s economy, people’s basic living needs are gradually met, and the high-level needs such as a good living environment have been put forward [1]. For example, the survey on Environmental Protection Awareness of Urban Residents in China in 2016 published by the Research Center of Public Opinion of Shanghai Jiaotong University shows that most urban residents are strongly dissatisfied with environmental pollution and regard environmental pollution as the most important problem to be solved by the government, and willing to contribute to improving the environmental quality. The report of the Nineteenth National Congress of the Communist Party of China emphasizes the construction of an environmental governance system with government as the leading factor, enterprises as the main body, social organizations, and public participation.

Despite the growing enthusiasm for public participation in environmental protection, the government still plays a leading role in China’s current environmental governance system. In many aspects, such as the formulation and adjustment of environmental protection policies, environmental law enforcement, environmental regulation and so on, the government still bears the primary responsibility. However, the single dominant governance model is difficult to effectively solve environmental problems, and the environmental governance has been confronted with the double dilemma of “government failure” and “market failure” [2]. Therefore, there are questions to clarify: Does public participation effectively improve the level of environmental governance? What is the relationship among government, enterprises, and the public in environmental governance? What is the impact mechanism of government supervision and public participation on enterprises’ environmental behavior?

This paper addresses these questions. The remainder of this research is organized as follows. In Section 2, we review relevant literature and identify the research gap that we focus on. Section 3 builds tripartite evolutionary game model including research hypothesis, parameters setting, and description, and pay-off matrix construction. In Section 4, replicated dynamic equation is established, the evolution process is analyzed, and stability analysis is carried out. In Section 5, based on China’s 30 provincial panel data from 2009 to 2018, we analyzed the impact of public participation and government supervision on enterprises’ pollution control behavior. Final conclusions and an outlook on future research direction are given in Section 6.

The contributions of this research are as follows. First of all, the current research literatures mostly explore the relationship between government and enterprises or between the public and enterprises, but do not put the government, enterprise, and the public in the same research framework. We regard enterprise, government, and the public as three bounded rational game players, and construct an evolutionary game model of government supervision, public participation and enterprise pollution control, and discuss the evolutionary process and stability strategy. 

Moreover, most literatures adopt traditional multiple regression model to study the influencing factors of enterprises’ environmental behavior, but neglect the trans-boundary characteristic of industrial pollution and spatial spillover effect. Based on the theory of spatial model, we build the spatial error model and spatial lag model, carry out further empirical research using 30 provincial panel data in China from 2009 to 2018, and provide suggestions for environmental governance from three perspectives of enterprise, government, and the public, which help to provide practical guidance and reference value.

## 2. Literature Review

With the development of economy, global environmental problems are becoming more and more serious. Global scholars have done a lot of research in the field of environmental governance. It mainly concentrates on two aspects: environmental governance mode and environmental governance model.

In traditional environmental governance, the “top-down” government-led mode is adopted, but now more attention has been paid to the bottom-up [3] and community-based model [4]. Khadka et al. (2012) uses both a top-down (TD) and a bottom-up (BU) approach to discuss how the two approaches have worked to incorporate the different views, opinions, and experiences of experts and stakeholders in sustainable forest management [5]. Reinsberger et al. (2015) point out that bottom-up initiatives are social innovations, which entail civil engagement in energy transition at a local or regional level, and are expected to play a growing role in the governance of local energy systems in Europe [6]. Serra-Llobet et al. (2016) compare how IWRM and flood risk management have been operationalized within “top-down” and “bottom-up” governance arrangements in the European Union and the United States, results show that the San Francisco Bay Area’s strongly collaborative and participatory approach has generated new connections among flood managers and other stakeholders [7]. Carolus et al. (2018) investigate the possible advantages, prerequisites and limitations of applying CBA (cost-benefit analysis) in what may be considered as an alternative, “bottom-up” manner [8]. Mohamad et al. (2015) explored the case for using “community-based shared values” as a potential driver for the “Heartware” aspects of governance in integrated watershed management (IWM)—from a Japan–Malaysia policy learning perspective [9]. Chen et al. (2016) examined the environmental challenges in the Caribbean islands, and discusses the regional inter-governmental approach and community-based local solutions, they advocate a polycentric governance approach which undertake a “local action, global impact” to achieve sustainable development [10]. Delgado-Serrano et al. (2018) point out that different social-ecological systems around the world are managed under community-based natural resource management (CBNRM) strategies, they analyze how CBNRM strategies influence the resilience of social-ecological systems to the disturbances they faced [11].

Bottom-up and community-based approaches emphasize the importance of public participation, so scholars have carried out research on public participation and multilateral cooperation. van der Vegt (2018) point out greater public engagement have emerged in policy circles and academia, particularly when it comes to risk-related decision-making, or risk governance over the last two decades [12]. Klinke (2012) designs new structures and processes of public deliberation and participation in trans-boundary regional environmental governance and develops a normative-analytical design for regional environmental governance in ecoregions [13]. Teng et al. (2013) analyze the environmental governance behaviors of the government and the enterprise. The Nash equilibrium is discussed under three different cases. Results show that if the government has a relatively large budget, it will satisfy its preference and the enterprise contribute nothing; if the government has a moderate budget, it will contribute all to the environmental governance while the enterprise still contribute nothing [14]. Cent et al. (2014) present an analysis of a consultation program conducted in the final stage of site selection for Natura 2000 in Malopolska. They analyze the modes and degrees of participation, the normative foundations of the consultation program, and the goals and expectations that characterize participants. The results show the limited success of the participatory process in representing all relevant stakeholders and enabling their actual influence on final decisions [15]. Paloniemi et al. (2015) explore public participation in biodiversity governance that has emerged after the initial designation of a Natura 2000 network in Finland, Greece, Poland, and the United Kingdom. They show that new participatory arrangements have taken the modes of project-based, market, interest group and e-governance, and call for public participation and wider stakeholder engagement in conservation [16]. Kochskamper et al. (2016) report on implementation of the WFD (European Water Framework Directive) in three case studies from Germany, Spain, and the United Kingdom. Results show that local participation improve the environmental standard of outputs and the quality of implementation [17]. Tan et al. (2017) employ a mixed methods approach with a qualitative emphasis to explore the process of communication and interaction between government agencies and local residents in Shandong Province, China. The results show that information disclosure of pollution data remains far from being transparent, despite the fact that the local government has implemented digital environmental governance, as encouraged by the central Chinese state [18]. Fernandez (2017) examines and explains the responses of Chilean municipalities and the role of municipal commitments and social capital. This article contributes to the Latin American literature about environmental management and disaster risk reduction at the local level [19]. Bodin (2017) points out collaborative governance is commonly put forward as the preferred means of addressing environmental problems. Under this paradigm, a deeper understanding of if, when, and how collaboration is effective, and when other means of addressing environmental problems are better suited [20]. Das (2019) aims to develop a framework incorporating public participation as a mandatory clause in water supply projects. The framework is developed to achieve effective public participation in six levels viz. inform, educate, consult, involve, collaborate, and capacity building [21]. Hensengerth et al. (2019) point out public participation legislation has suffered from an implementation gap, leading to the proliferation of environmental protests across China. There are indications that protests may result in the improvement and creation of local institutions that facilitate public participation, which in turn help to foster a new model of governance that contains features of multi-level governance [22].

At the same time, scholars have carried out quantitative research and built many models of environmental governance. Wang et al. (2015) analyze the interest relationship between the enterprise and the public in environmental governance by game method. Two complete information dynamic game models are established to descript the choices of strategy for enterprises. The game equilibrium shows that the public power has important influence on the choice of strategy for enterprise [23]. Plummer et al. (2017) confirm a model showing that participation in more activities leads to greater ratings of process, and in turn, better evaluations of outcomes. Original insights are offered as to how the evaluations of outcomes by stakeholders are shaped by their participation in activities and their experiences in management and governance processes [24]. Zhang et al. (2018) put forward a theoretical model illustrating the significance of public participation. They build an empirical model to investigate the impact of public participation on pollutant emissions of the four main pollutants SO_2_, NOx, COD, and NH4, based on the panel data of 30 provinces from 2011 to 2015 in China [25]. Gao et al. (2018) evaluate the performance of urban environmental governance by developing hesitant fuzzy linguistic analytic network process (HFL-ANP). Through three urban cases of environmental governance, HFL-ANP is proved to be a very suitable method of assessing environmental governance [26]. Zheng et al. (2018) build a stochastic frontier analysis (SFA) model to estimate the input efficiency of environmental governance in China, explore the overall characteristics of input efficiency, and explore the evolving trends in terms of the degree of match between environmental input efficiency and input efficiency in various provinces [27].

All above-mentioned literatures provide direction for theoretical and practical research in environmental governance. However, most of the studies are static qualitative analysis and social investigation methods, and focus on issues such as the positive role of public participation, the influencing factors of public participation and the ways of public participation, and lack of research on the interaction among the public, enterprises and the government. Only a few scholars have studied about how much the balanced relationship between participants can contribute to reducing the risk of disasters [28,29,30,31], therefore, the relationship between participants needs to be further studied. The environmental governance is essentially a game process of related participants. The interaction process of participants should be analyzed from a dynamic perspective. Most literature studies the game between government and enterprise, the public and enterprise, and don’t put the government, enterprise, and public in the same research framework. We build a tripartite evolutionary game model of government, public and enterprise, and analyze the interaction mechanism among them, and adopt 30 provincial panel data of China from 2009 to 2018 to make an empirical test. 

## 3. Construction of Evolutionary Game Model

We propose the following three hypotheses.

(1) Government, enterprises, and the public are bounded rational. Because of information asymmetry, they have limited abilities in rational cognition, analytical reasoning, and decision-making [32].

(2) Assuming that the local economic development originates from the production activities and value creation of enterprises, the economic benefits of enterprises can reflect the local economic development level. Pollution discharge by enterprises is the main source of environmental pollution, and the environmental benefits brought by enterprises’ environmental behaviors can reflect the local environmental quality.

(3) According to the environmental quality, the government determines the intensity of enterprise supervision and the public participation incentives.

**Figure 1:**
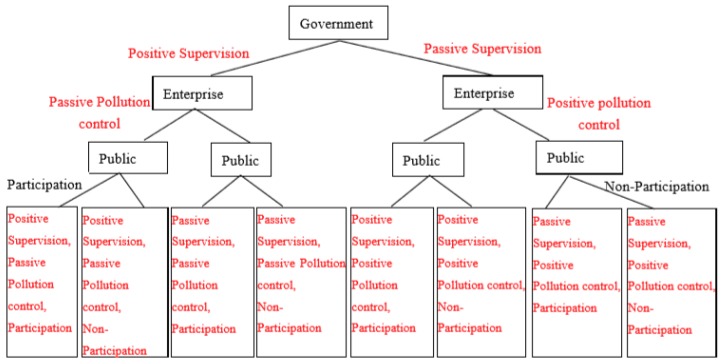
Tripartite game strategies.

The tripartite game strategies combination of government, enterprise and the public are shown in Figure 1.

(1) Enterprises. The strategy set of enterprise is S_1_ = {positive pollution control, passive pollution control}. “Positive pollution control” means that enterprises control pollution through adopting environmental protection facilities or changing production modes. The enterprises declare to the local government the type, quantity, concentration, destination and mode of pollutants and reach the pollution discharge standard. “Passive pollution control” means that enterprises do a little or no pollution treatment which result in unqualified pollutant discharge. Based on the assumption of bounded rationality, without supervision, enterprises will not bear the penalty cost of unqualified pollutant discharge. Therefore, in order to maximize profits, enterprises will choose low-cost “passive pollution control” strategy. In the case of positive supervision, if enterprises choose “positive pollution control” strategy, they will increase their production costs, and if they choose “passive pollution control” strategy, they will bear the penalty costs. Therefore, the main factor affecting the dominant strategy is the difference between production cost and penalty cost, namely its marginal cost. Assuming that the probability of the enterprises choosing “positive pollution control” strategy is *x*, *x* ∈ [0,1], the probability of “passive pollution control” strategy is 1 − *x*.

(2) Government. The strategy set of government is S_2_ = {positive supervision, passive supervision}. “Positive supervision” means that the government invests manpower, material resources, and financial resources to supervise and manage the enterprises’ environmental behavior. It includes not only environmental subsidies and administrative penalties for enterprises, but also various incentives for public participation. “Passive supervision” means the government does not intend to intervene and punish enterprises’ environmental behaviors such as unqualified pollutant discharge, secretly emissions, leakage emissions, etc. Strict supervision leads to difficulties in the development of enterprises and affects the performance of local governments, so the government has the possibility of passive supervision. Without positive supervision, it is unrealistic to prevent enterprises from polluting the environment only by self-restraint mechanism and market economy measures. Therefore, “positive supervision” is the dominant strategy for the government. Assuming that the probability of the government choosing “positive supervision” strategy is y, y ∈ [0,1], the probability of "passive supervision" strategy is 1 − y.

(3) The public. The strategy set of the public is S_3_ = {participation, non-participation}. “Participation” means that the public supervise the enterprises’ environmental pollution behaviors by means of reporting, petitioning, exposure, litigation and claim for compensation, etc. “Non-participation” means that the public ignores the enterprises’ environmental pollution behaviors. Assuming that the probability of public choosing "participation" strategy is *z*, *z* ∈ [0,1], and the probability of “non-participation” strategy is 1 − *z*.

In order to study the cost, benefit, and loss of government, enterprise, and the public, we need to set relevant parameters. According to the relevant provisions of laws and regulations such as Environmental Protection Law of the People’s Republic of China, Water Pollution Prevention Law of the People’s Republic of China, Air Pollution Prevention Law of the People’s Republic of China, Solid Waste Pollution Prevention Law of the People’s Republic of China and Measures for Public Participation in Environmental Protection, the parameters are defined and described as shown in Table 1. 

Based on the above parameters, we can get the pay-off matrix, as shown in Table 2.

## 4. Analysis of Evolutionary Game Model

### 4.1. Replicated Dynamic Equation and Evolutionary Stabilization Strategy

In evolutionary game, enterprises, government and the public are all decision-makers with limited rationality, so the optimal strategy usually can’t be found immediately in the initial stage. The best strategy will be found through trial and error in the continuous game. At the same time, the equilibrium is not the result of one-time choice, but result of constant adjustment and improvement, even if it reaches the equilibrium, it may deviate again [33]. Therefore, in order to effectively analyze and predict the game among enterprises, government and the public, it is necessary to adopt a suitable analysis method. The “replication dynamics” mechanism can well simulate the learning and dynamic adjustment process, and the “evolutionary stability strategy” is suitable for analyzing the stability and dynamic development trend. We construct replicated dynamic equations, and analyze their evolutionary stabilization strategies.

Assuming that the expected return of enterprise choosing “positive pollution control” strategy is *E*_11_, the expected return of enterprise choosing “passive pollution control” strategy is *E*_12_, the expected return of government choosing “positive supervision” strategy is *E*_21_, the expected return of government choosing “passive supervision” strategy is *E_22_*, the expected return of public choosing “participation” strategy is *E*_31_, and the public choosing “non-participation” strategy is *E*_32_. $\bar{E_{1}}$, $\bar{E_{2}}$, and $\bar{E_{3}}$ are the average expected returns of enterprise, government, and the public respectively. Equation (1)–(3) is given as follows:

(1) $$\bar{E_{1}}=\;x\;E_{11}+(1-x)E_{12}\;=x(yS+zI_{e3}+I_{e2}-C_{e}-(1-X)(I_{e1}-yF-zpL_{e})$$

(2) $$\bar{E_{2}}=y\;E_{21}+(1-y)\;E_{22}\;=y[x(I_{g}-S)-(1-x)(F-C_{g2})-zR-C_{g1}]+(1-y)[xI_{g}-(1-x)I_{g2}]$$

(3) $$\bar{E_{3}}=z\;E_{31}+(1-z)\;E_{32}\;=z[x(I_{p2}+L_{p})+yR+I_{p1}-C_{p}-L_{p}]+(1-z)[x(I_{p2}+L_{p})-L_{p}]$$

The replicated dynamic equation of enterprise positive pollution control, government positive supervision and public participation can be given in Equation (4):

(4) $$F(x)=\frac{d_{x}}{d_{t}}=x(E_{11}-\bar{E_{1}})=x(1-x)[z(Ie_{3}+pL_{e})+y(S+F)+Ie_{2}-C_{e}-Ie_{1}]\;F(y)=\frac{d_{y}}{d_{t}}=y(E_{21}-\bar{E_{2}})=y(1-y)[x(F-S-2Cg_{2})-zR-F+2Cg_{2}-Cg_{1}]\;F(z)=\frac{d_{z}}{d_{t}}=z(E_{31}-\bar{E_{3}})=z(1-z)(yR+xIp_{2}+Ip_{1}-C_{p})$$

The following is a detailed analysis of the replicated dynamic equation and the evolutionary stabilization strategy. According to Equation (4), we know that replicated dynamic equation of enterprise positive pollution control is Equation (5), as follows:

(5) $$F(x)=\frac{d_{x}}{d_{t}}=x(E_{11}-\bar{E_{1}})=x(1-x)[z(Ie_{3}+pL_{e})+y(S+F)+I_{e2}-C_{e}-I_{e1}]$$

Derivative of *F*(*x*) is given in Equation (6):

(6) $$F^{\prime }(x)=\frac{dF(x)}{d_{x}}=(1-2x)[z(I_{e3}+pL_{e})+y(S+F)+I_{e2}-C_{e}-I_{e1}]$$

The evolutionary stabilization strategies of enterprises’ positive pollution control are analyzed as follows.

Proposition 1. The probability of enterprises adopting “positive pollution control” strategy will increase with the probability increase of government adopting “positive supervision” strategy.

Demonstration: When y > $\frac{I_{e1}+C_{e}-I_{e2}-z(I_{e3}+pL_{e})}{S+F}$, ${\frac{dF(x)}{d_{x}}|}_{x=1}$ < 0, ${\frac{dF(x)}{d_{x}}|}_{x=0}$ > 0, then *x* = 1 is an evolutionary stabilization strategy. When y < $\frac{I_{e1}+C_{e}-I_{e2}-z(I_{e3}+pL_{e})}{S+F}$, ${\frac{dF(x)}{d_{x}}|}_{x=1}$ > 0, ${\frac{dF(x)}{d_{x}}|}_{x=0}$ < 0, then *x* = 0 is an evolutionary stabilization strategy. The probability of enterprises adopting “positive pollution control” strategy will increase with the probability increase of government adopting “positive supervision” strategy. It shows that the government positive supervision and management can urge enterprises to carry out positive pollution control.

Proposition 2. The probability of enterprises adopting “positive pollution control” strategy will increase with the probability increase of public adopting “participation” strategy.

Demonstration: When z > $\frac{I_{e1}+C_{e}-I_{e2}-y(S+F)}{I_{e3}+pL_{e}}$, ${\frac{dF(x)}{d_{x}}|}_{x=1}$ < 0, ${\frac{dF(x)}{d_{x}}|}_{x=0}$ > 0, then *x* = 1 is an evolutionary stabilization strategy. When z < $\frac{I_{e1}+C_{e}-I_{e2}-y(S+F)}{I_{e3}+pL_{e}}$, ${\frac{dF(x)}{d_{x}}|}_{x=1}$ > 0, ${\frac{dF(x)}{d_{x}}|}_{x=0}$ < 0, then *x* = 0 is an evolutionary stabilization strategy. The probability of enterprises adopting “positive pollution control” strategy will increase with the probability increase of public adopting “participation” strategy. It shows that the public participation in environmental governance can urge enterprises to carry out positive pollution control.

Proposition 3. The probability of enterprises adopting “positive pollution control” strategy depends on the enterprise’s initial return, pollution control cost, and reputation benefits. The effect of government supervision is constrained by the income and cost of enterprises.

**Figure 2:**
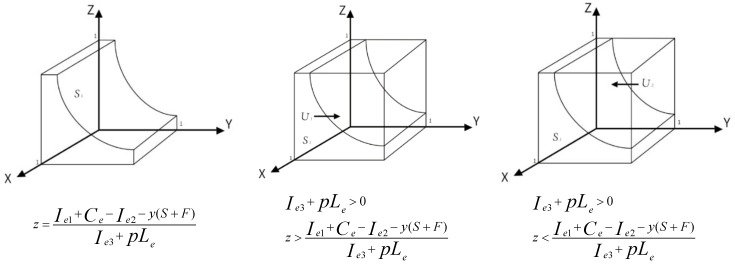
Trend chart of enterprise evolution.

Demonstration: The trend of evolutionary stabilization strategy for enterprise is shown in Figure 2. 

*U*_1_ and *U*_2_ are the probabilities of enterprises adopting “positive pollution control” strategy and “passive pollution control” strategy respectively. z = $\frac{I_{e1}+C_{e}-I_{e2}-y(S+F)}{I_{e3}+pL_{e}}$, if *z* = 0, then y = $\frac{I_{e1}+C_{e}-I_{e2}}{S+F}$, if *z* = 1, then y = $\frac{I_{e1}+C_{e}-I_{e2}-I_{e3}-pL_{e}}{S+F}$. *U*_1_ = 1 − *U*_2_ = 1 − $\frac{2I_{e1}+2C_{e}-2I_{e2}-I_{e3}-pL_{e}}{2S+2F}$.

When $\frac{dU_{1}}{dC_{e}}=\frac{-2}{2S+2F}<0$, $\frac{dU_{1}}{dI_{e1}}=\frac{-2}{2S+2F}<0$, it means that the higher the enterprise benefits without positive pollution control or the higher the pollution control cost, the smaller the probability of enterprises adopting “positive pollution control” strategy. When $\frac{{dU}_{1}}{{dI}_{e2}}=\frac{2}{2S+2F}>0$, $\frac{{dU}_{1}}{{dI}_{e3}}=\frac{1}{2S+2F}>0$,$\frac{{dU}_{1}}{{dL}_{e}}=\frac{p}{2S+2F}>0$, it means that if the enterprise adopts “positive pollution control” strategy, it will get more initial return and reputation benefits. If the enterprise adopts “passive pollution control” strategy, it will have huge reputation loss, and the possibility of the enterprise choosing “positive pollution control” strategy becomes higher.

When *I*_*e*1_ < *I*_*e*2_ − *C_e_*, then $\frac{{dU}_{1}}{dF}=\frac{{dU}_{1}}{dS}=\frac{2I_{e1}+2C_{e}-2I_{e2}-I_{e3}-pL_{e}}{{\left(2S+2F\right)}^{2}}<0$. It means that the probability of the enterprise adopting “positive pollution control” strategy will decrease with the strengthening of government supervision, when the benefits of "passive pollution control" strategy are less than those of “positive pollution control” strategy. When *I*_*e*1_ > *I*_*e*2_ − *C_e_*, then $\frac{{dU}_{1}}{dF}=\frac{{dU}_{1}}{dS}=\frac{2I_{e1}+2C_{e}-2I_{e2}-I_{e3}-pL_{e}}{{\left(2S+2F\right)}^{2}}>0$. It means that the probability of enterprise adopting “positive pollution control” strategy will increase with the strengthening of government supervision, when the benefits of “passive pollution control” strategy are greater than those of “positive pollution control” strategy.

In the same way, the following propositions can be drawn by constructing and solving replicated dynamic equations of “government positive supervision” and “public participation” and analyzing their evolutionary stabilization strategies.

Proposition 4. The probability of government adopting “positive supervision” strategy will decrease with the probability increase of enterprises adopting “positive pollution control” strategy.

Proposition 5. The probability of government adopting “positive supervision” strategy will decrease with the probability increase of public adopting “participation” strategy.

Proposition 6. The probability of government adopting “positive supervision” strategy will decrease with the increase of government supervision cost.

Proposition 7. The probability of the public adopting “participation” strategy will increase with the probability increase of government adopting “positive supervision” strategy.

Proposition 8. The probability of the public adopting “participation” strategy will decrease with the increase of the public participation cost, and increase with the increase of psychological benefits.

### 4.2. Equilibrium Point and Stability Analysis

By analyzing the tripartite evolutionary game model among enterprises, government, and the public, we can get eight equilibrium points are shown in Table 3.

(1) If *x* = 0, *y* = 0, then *yR + xI_p2_* + *I*_*p*1_ − *C_p_* = *I*_*p*1_ − *C_p_*. If *I*_*p*1_ < *C_p_*, then evolutionary equilibrium stability point is (*x* = 0, *y* = 0, *z =* 0). Namely, if the enterprise passive pollution control (*x* = 0), and the government passive supervision (*y* = 0), which means that the environmental quality will not be improved, and public will not get rewards from government, then the public will choose “non-participation” when psychological benefits are less than participation costs, so the evolutionary stability strategy is (passive pollution control, passive supervision, non-participation). Else, if *I*_*p*1_ > *C_p_*, then evolutionary equilibrium stability point is (*x* = 0, *y* = 0, *z* = 1). Namely, the public will choose “participation” when psychological benefits are greater than participation costs, so the evolutionary stability strategy is (passive pollution control, passive supervision, participation).
(2) If *x* = 1, *y* = 0, then *yR + xI*_*p*2_ + *I*_*p*1_ − *C_p_* = *I*_*p*2_ + *I*_*p*1_ − *C_p_*. If *I*_*p*2_ + *I*_*p*1_ < *C_p_*, then evolutionary equilibrium stability point is (*x* = 1, *y* = 0, *z* = 0). Namely, if the enterprise positive pollution control (*x* = 1), and the government passive supervision (*y* = 0), which means that the environmental quality will be improved, public will not get rewards from government, then the public will choose “non-participation” when the sum of psychological benefits and environmental benefits are less than participation costs, so the evolutionary stability strategy is (positive pollution control, passive supervision, non-participation). Else, if *I*_*p*2_ + *I*_*p*1_ > *C_p_*, then evolutionary equilibrium stability point is (*x* = 1, *y* = 0, *z* = 1). Namely, the public will choose “participation” when the sum of psychological benefits and environmental benefits are greater than participation costs, so the evolutionary stability strategy is (positive pollution control, passive supervision, participation).
(3) If *x* = 0, *y* = 1, then *yR + xI*_*p*2_ + *I*_*p*1_ − *C_p_* = *R* + *I*_*p*1_ − *C_p_*. If *R* + *I*_*p*1_ < *C_p_*, then evolutionary equilibrium stability point is (*x* = 0, *y* = 1, *z* = 0). Namely, if the enterprise passive pollution control (*x* = 0), and the government positive supervision (*y* = 1), which means that the environmental quality will not be improved, public will get rewards from government, then the public will choose “non-participation” when the sum of the government rewards and psychological benefits are less than participation costs, so the evolutionary stability strategy is (passive pollution control, positive supervision, non-participation). Else if *R* + *I*_*p*1_ > *C_p_*, then evolutionary equilibrium stability point is (*x* = 0, *y* = 1, *z* = 1). Namely, the public will choose “participation” when the sum of government rewards and psychological benefits are greater than participation costs, so the evolutionary stability strategy is (passive pollution control, positive supervision, participation).
(4) If *x* = 1, *y* = 1, then *yR + xI*_*p*2_ + *I*_*p*1_ − *C_p_* = *R* + *I*_*p*2_ + *I*_*p*1_ − *C_p_*. If *R* + *I*_*p*2_ + *I*_*p*1_ < *C_p_*, then evolutionary equilibrium stability point is (*x* = 1, *y* = 1, *z* = 0). Namely, if the enterprise positive pollution control(*x* = 1), and the government positive supervision(*y* = 1), which means that the environmental quality will be improved, and public will get rewards from government, then the public will choose “non-participation” when the sum of government rewards, psychological benefits and environmental benefits are less than participation costs, so the evolutionary stability strategy is(Positive Pollution control, Positive Supervision, Non-Participation). Else if *R* + *I*_*p*2_ + *I*_*p*1_ > *C_p_*,, hen evolutionary equilibrium stability point is(*x* = 1, *y* = 1, *z* = 1). Namely, the public will choose “participation” when the sum of government rewards, psychological benefits and environmental benefits are greater than participation costs, so the evolutionary stability strategy is (Positive Pollution control, Positive Supervision, Participation).

To achieve the goal of environmental governance, enterprises should positive pollution control, government should positive supervision and public should participation, so the ideal state is x = 1, y = 1, z = 1). By controlling or adjusting the relevant variables, the evolutionary strategy can evolve to the ideal state.

When z > $\frac{I_{e1}+C_{e}-I_{e2}-y(S+F)}{I_{e3}+pL_{e}}$, then x = 1. That is, the enterprise chooses “positive pollution control”. Therefore, there are some factors affect enterprise to positive pollution control: reduce the pollution control cost of enterprises, decrease profits if enterprise passive pollution control, increase profits if enterprise positive pollution control, strengthen government supervision, increase the punishment to enterprises and the rewards to the public, give praise and motivation to enterprises for positive pollution control, the more reputational benefits an enterprise gains, the more willing it is to positive pollution control, enlarge the reputation loss if enterprises passive pollution control and improve the probability of public discover and report enterprise.

When $z>\frac{x(F-S-2C_{g2})-F+2C_{g2}-C_{g1}}{R}$, then y = 1. That is, the government chooses “positive supervision”. Therefore, there are some factors affect government to positive supervision: reduce the cost of human, material and financial resources, increase penalties for passive pollution control enterprises and subsidies for positive pollution control enterprises, and raise the rewards for public participation.

When $x>\frac{C_{p}-I_{p1}-yR}{I_{p2}}$, then z = 1. That is, the public chooses “participation”. Therefore, there are some factors affect public to participation: reduce the participation cost, and increase psychological benefits, environmental benefits and government rewards. 

Evolutionary game analysis shows that both government supervision and public participation will have a positive impact on the enterprise environmental behavior. If enterprises pollute the environment, they may face severe administrative penalties and enormous pressure of public opinion. Government supervision and public participation will influence each other. On the one hand, public participation can restrain the improper behavior of the government. On the other hand, through legal system and public opinion propaganda, the government regulates the ways of public participation, so as to promote the efficient development of environmental protection work. 

Environmental governance needs to mobilize the whole society, give full play to the role of the government, the public, and enterprises. Integrate resources and have complementary advantages, and adopt a combination of ”op-down” nd “ottom-up” approaches to promote the ultimate realization of ecological civilization in a stable, orderly, and powerful way. First, insist on the dominant position of the government. The key is to strengthen the top-level design of environmental governance system, that is, to achieve institutional innovation on the basis of green integration of existing ecological environment-related policies. Second, the government guides and guarantees public participation legally and orderly. The development of environmental NGOs can be seen as a reflection of the strategy of ”mall government, big society” is not only the result of the government’s top-down efforts to protect the ecological environment, but also the result of the public’s bottom-up efforts to obtain the right of speech. Finally, give play to the key role of enterprises. Enterprises should change development ideas. Protecting the environment and dealing with pollution no longer means an expensive burden, but a means to enhance the competitiveness of enterprises. At the same time, enterprises try to realize the ecological transformation, that is, from the extensive production mode with high input, high consumption, high pollution and low efficiency to the resource-saving and environment-friendly production mode.

## 5. Empirical Test

### 5.1. Variable Description and Model Construction

The definition and description of variables are shown in Table 4.

According to the viewpoint of space economics, there is a spatial connection in the development of things, and the development of things will affect each other [38]. Air pollution, water pollution, solid waste pollution, and other major environmental pollutants have the characteristics of diffusion, mobility, and transmission. Environmental pollution has obvious spatial spillover effect. The current literatures usually adopt traditional multiple regression models to study the influencing factors of enterprises’ environmental behavior, but ignore the trans-boundary characteristic and spatial spillover effects of industrial pollution. In this paper, according to the spatial model theory, the spatial lag model and spatial error model are built on the basis of the traditional econometric model, and 30 provincial panel data in China from 2009 to 2018 are collected to analyze the impact of government supervision and public participation on enterprises’ environmental behavior. The large likelihood method is used to solve the model.

The traditional econometric model is given in Equations (7), (8) and (9) as follows, which express the impact of government supervision and public participation on enterprise environmental behavior.

(7) $${Iwg}_{i,t}=\alpha_{0}+\alpha_{1}{Gwg}_{i,t}+\alpha_{2}{Pwg}_{i,t}+\alpha_{3}{Gwg}_{i,t}\times {Pwg}_{i,t}+\alpha_{4}{Psg}_{i,t}+\alpha_{5}{Neu}_{i,t}+\alpha_{6}{Dpe}_{i,t}+\mu_{i,t}$$

(8) $${Iww}_{i,t}=\beta_{0}+\beta_{1}{Gww}_{i,t}+\beta_{2}{Pww}_{i,t}+\beta_{3}{Gww}_{i,t}\times {Pww}_{i,t}+\beta_{4}{Psg}_{i,t}+\beta_{5}{Neu}_{i,t}+\beta_{6}{Dpe}_{i,t}+\mu_{i,t}$$

(9) $${Isw}_{i,t}=\gamma_{0}+\gamma_{1}{Gsw}_{i,t}+\gamma_{2}{Psw}_{i,t}+\gamma_{3}{Gsw}_{i,t}\times {Psw}_{i,t}+\gamma_{4}{Psg}_{i,t}+\gamma_{5}{Neu}_{i,t}+\gamma_{6}{Dpe}_{i,t}+\mu_{i,t}$$

Where, *i* is provinces, municipalities and autonomous regions, (*i* = 1, 2, …, 30), *t* is year (*t* = 2009, 2010, …, 2018), *α_i_*, *β_i_* and $\gamma_{i}$ are regression coefficients, and $\mu_{i,t}$ is error term. Equations (7), (8), and (9) are the influencing factor models of industrial waste gas, industrial waste water, and industrial solid waste respectively.

Considering the trans-boundary characteristic and the spatial spillover effect can be represented by lag term and error term, we build spatial lag model and spatial error model respectively on the basis of Equations (7), (8), and (9). Because both spatial lag model and spatial error model have spatial autocorrelation, the results obtained by the least squares estimation method may be biased or invalid. Therefore, the maximum likelihood estimation method is used to solve the spatial lag model and the spatial error model.

(1) Construction of Spatial Lag Model 

Based on Equations (7), (8), and (9), we introduce the spatial variables, and decompose the random error term $\mu_{i,t}$ into time-effect stochastic perturbation term $\delta_{i,t}$, individual-effect stochastic perturbation term $\xi_{i,t}$ and random error term $\varepsilon_{i,t}$. The spatial lag model is given in Equations (10), (11), and (12) as follows.

(10) $${Iwg}_{i,t}=\alpha_{0}+\alpha_{1}{Gwg}_{i,t}+\alpha_{2}{Pwg}_{i,t}+\alpha_{3}{Gwg}_{i,t}\times {Pwg}_{i,t}+\alpha_{4}{Psg}_{i,t}+\alpha_{5}{Neu}_{i,t}+\;\alpha_{6}{Dpe}_{i,t}+\varphi \sum W{Iwg}_{i,t}+\delta_{i,t}+\xi_{i,t}+\varepsilon_{i,t}$$

(11) $${Iww}_{i,t}=\beta_{0}+\beta_{1}{Gww}_{i,t}+\beta_{2}{Pww}_{i,t}+\beta_{3}{Gww}_{i,t}\times {Pww}_{i,t}+\beta_{4}{Psg}_{i,t}+\beta_{5}{Neu}_{i,t}+\beta_{6}{Dpe}_{i,t}+\;\varphi \sum W{Iww}_{i,t}+\delta_{i,t}+\xi_{i,t}+\varepsilon_{i,t}$$

(12) $${Isw}_{i,t}=\gamma_{0}+\gamma_{1}{Gsw}_{i,t}+\gamma_{2}{Psw}_{i,t}+\gamma_{3}{Gsw}_{i,t}\times {Psw}_{i,t}+\gamma_{4}{Psg}_{i,t}+\gamma_{5}{Neu}_{i,t}+\gamma_{6}{Dpe}_{i,t}+\;\varphi \sum W{Isw}_{i,t}+\delta_{i,t}+\xi_{i,t}+\varepsilon_{i,t}$$

Where, φ is spatial autoregressive coefficient, *W* is spatial weighted matrixes, $\varepsilon_{i,t}∼N(0,\sigma_{i,t}^{2})$.

(2) Construction of Spatial Error Model

If other factors with spatial properties are considered in random error item $\varepsilon_{i,t}$, then the random error term may have strong correlation and is no longer normal distribution. So the spatial error model is given in Equations (13), (14), and (15) as follows.

(13) $${Iwg}_{i,t}=\alpha_{0}+\alpha_{1}{Gwg}_{i,t}+\alpha_{2}{Pwg}_{i,t}+\alpha_{3}{Gwg}_{i,t}\times {Pwg}_{i,t}+\alpha_{4}{Psg}_{i,t}+\alpha_{5}{Neu}_{i,t}+\;\alpha_{6}{Dpe}_{i,t}+\delta_{i,t}+\xi_{i,t}+\varepsilon_{i,t}$$

(14) $${Iww}_{i,t}=\beta_{0}+\beta_{1}{Gww}_{i,t}+\beta_{2}{Pww}_{i,t}+\beta_{3}{Gww}_{i,t}\times {Pww}_{i,t}+\beta_{4}{Psg}_{i,t}+\beta_{5}{Neu}_{i,t}+\;\beta_{6}{Dpe}_{i,t}+\delta_{i,t}+\xi_{i,t}+\varepsilon_{i,t}$$

(15) $${Isw}_{i,t}=\gamma_{0}+\gamma_{1}{Gsw}_{i,t}+\gamma_{2}{Psw}_{i,t}+\gamma_{3}{Gsw}_{i,t}\times {Psw}_{i,t}+\gamma_{4}{Psg}_{i,t}+\gamma_{5}{Neu}_{i,t}+\gamma_{6}{Dpe}_{i,t}+\;\delta_{i,t}+\xi_{i,t}+\varepsilon_{i,t}$$

Where $\varepsilon_{i,t}=\rho \Sigma W_{\varepsilon_{i,t}}+\nu_{i,t}$, $\nu_{i,t}∼N(0,\sigma_{i,t}^{2})$, *ρ* is spatial correlation coefficient of regression residuals.

### 5.2. Data Source 

The sample of this study covers 30 provinces, municipalities, and autonomous regions in China. The sample time span is 2009–2018. In order to obtain typical provincial environmental panel data in China, the following principles are adopted in this study: (a) In order to ensure the timeliness and foresight of the research, the data deadline of this study is 2018, and the latest environmental data of 2018 released by the Ministry of Ecology and Environment. (b) Considering the special geographical location of Xinjiang and Tibet, and missing sample data in some years, this study excludes them.

The data in this study are mainly from China Environmental Statistics Yearbook, China Environmental Yearbook, National Environmental Statistics Bulletin, China Civil Affairs Statistics Yearbook, China Population Statistics Yearbook, and government websites, and portal sites.

### 5.3. Analysis of Empirical Results

We use MATLAB to calculate the panel data. The result is given in Table 5. The Lagrange factor test results of spatial lag model and spatial error model show that both models have passed the significance test and robustness test. By comparing the coefficient of determination, parameter significance level, and log likelihood ratio of the two models, we choose the spatial lag model with better goodness-of-fit and significance for further discussion and analysis.

From Table 5, we can see that government supervision is negatively related to enterprise pollution control. The regression coefficients of industrial waste gas, industrial waste water, and industrial solid waste are negative and significant at the confidence levels of 5%, 5%, and 10%, respectively. It shows that with the strengthening of the government supervision, the cost of enterprises’ illegal discharge increases, and enterprises may even face severe administrative penalties. Therefore, enterprises will choose to actively control pollution with the affordable cost. The effect of government supervision is also affected by the cost and benefits of enterprise’s pollution control. When enterprises are faced with environmental regulation, they can choose to shut down, move or reduce production. They can also choose to invest funds to improve production technology or to develop green production technology. It depends on which strategy can maximize the economic benefits of enterprises. With the enhancement of environmental protection awareness, enterprises actively perform environmental governance obligation, the government will reduce environmental regulation in order to reduce the cost of supervision. Improving the reputation loss can effectively stimulate local government’s environmental supervision behavior.

Public participation is negatively related to enterprise pollution control. The regression coefficients of industrial waste gas, industrial waste water, and industrial solid waste are negative and significant at the confidence levels of 5%, 10%, and 10% respectively. It shows that public participation can significantly affect the discharge of three industrial wastes. With the increase of public participation, the discharge of three wastes shows a downward trend. Public participation is closely related to the cost and psychological benefits. When the public’s sense of achievement, identity and self-confidence is greater than the time cost, material cost and psychological cost, the public will actively participate in environmental governance. Public participation can not only effectively assist government supervision, but also improve the probability of discovering enterprises’ unqualified pollutant discharge, thus stimulating enterprises’ positive pollution control. 

The cross terms of public participation and government supervision are all negative. The regression coefficients of industrial waste gas, industrial waste water, and industrial solid waste are negative and significant at the confidence levels of 5%, 10%, and 5% respectively. It shows that public participation can replace the role of government supervision to a certain extent. To some extent, public participation can share the pressure of government supervision and promote the effect of environmental governance. Improve the ability of public participation, form a third-party environmental governance supervision mechanism, supervise the environmental behavior of government and enterprises, and solve the dilemma of “government failure” and “market failure.”

## 6. Conclusions

Based on the tripartite evolutionary game model and 30 provincial panel data in China, this paper analyses the relationship among government, enterprises, and the public in environmental governance, and points out the influencing factors of government and the public on enterprises’ environmental behavior. Based on the research results, the following suggestions are put forward from the perspective of government, enterprises, and the public.

(1) Government improves the ability of environmental regulation 

Through the mechanism of “propaganda-organization-supervision-punishment”, the government solves a series of difficult problems such as environmental regulation and environmental law enforcement. Popularize environment protection knowledge through network media and other ways, improve the environmental protection institutions, mechanisms and policies, improve environmental protection laws and regulations, refine environmental standards, and strengthen environmental supervision and bureaucratic accountability. Increase investment in environmental monitoring equipment, funds and personnel, mobilize public participation in the supervision of enterprises pollution behavior. Reward for reporting, establish electronic network channels for letters and visits, reduce the cost of public participation and improve the efficiency of participation. The government imposes economic fines and administrative penalties on enterprises’ passive pollution behavior, and gives subsidies to environmental protection behavior.

(2) Enterprises improve the ability of pollution control

Enterprises should strengthen their ability of scientific research and innovation, develop and master core technologies, accelerate the transformation of development mode, realize the effective transformation and upgrading of high energy consumption and high pollution industries, improve the efficiency of energy recycling, and reduce the discharge of various pollutants. At the same time, enterprises should strengthen deep cooperation with scientific research institutions, transform and absorb frontier technologies, upgrade green technology in non-environmental protection industries, and develop low-carbon economy and green financial model.

(3) Public improve the ability of participation

The public should positively exercise the right to know, the right to participate, the right to report, the right to claim compensation, and the right to sue. The public should develop the ability of independence and specialization, enhance authority and justice, strengthen cooperation with government and media, improve their management and organization level, develop the ability of horizontal communication and cultivate international vision, provide forward-looking policy recommendations for decision-making, and better play its role in environmental governance.

According to China’s basic national conditions, we need to adhere to the “top-down” government-led environmental governance. At the same time, we need to properly guide and give full play to the enterprises and the public, arouse their enthusiasm and great potential. Only by combining the “top-down” and “bottom-up” approaches, can we achieve ecological civilization.

As a complex system, government and public participation in enterprise environmental behavior are affected by many factors. We don’t consider the influencing factors comprehensively enough. Some influencing factors, such as relevant regulations and policies will be further studied in future research. 

## Figures and Tables

**Table 1:** Parameters and description.

Notation	Description
*I*_*e*1_	The initial return of enterprise who adopts “passive pollution control” strategy
*I*_*e*2_	The initial return of enterprise who adopts “positive pollution control” strategy
*I*_*e*3_	In the case of public participation, reputation benefits of enterprise for active pollution control
*I*_*p*1_	Psychological benefits of public for participation in environmental governance
*I*_*p*2_	If enterprise adopts “positive pollution control” strategy, then the public get the benefits of environmental improvement
*I_g_*	If enterprise adopts “positive pollution control” strategy, then the government get the potential benefits
*L_e_*	If enterprise adopts “passive pollution control” strategy, in the case of public participation, enterprises will lose reputation
*L_p_*	If enterprises adopt “passive pollution control” strategy, public will suffer from environmental pollution
*C_e_*	The pollution control cost of enterprise
*C*_*g*1_	If government adopts “positive supervision” strategy, the cost of human, material, and financial resources invested by the government
*C*_*g*2_	If enterprise adopts “passive pollution control” strategy which leads to pollution accidents, the cost of government handling accidents
C_p_	Participation cost of the public including information cost, opportunity cost, and sometimes infringement cost.
*R*	Rewards for public participation in environmental governance when government adopts “positive supervision” strategy.
*F*	If enterprise adopts “passive pollution control” strategy, in the case of government positive supervision, enterprise will be penalized by the government.
*S*	If the enterprise adopts “positive pollution control” strategy, in the case of government positive supervision, enterprises will get environmental protection subsidies given by the government.
*p*	The probability that enterprise adopts “passive pollution control” strategy and is reported by the public.

**Table 2:** Pay-off matrix of government, enterprise, and public.

Tripartite Game Strategy	Enterprise Payoff	Government Payoff	Public Payoff
(Positive Pollution control, Passive Supervision, Participation)	*I*_*e*1_ * − pL_e_ − F*	*F − C*_*g*1_ − *C*_*g*2_ − *R*	*I*_*p*1_ − *C_p_ + R − L_p_*
(Passive Pollution control, Positive Supervision, Non-Participation)	*I*_*e*1_ * − F*	*F − C*_*g*1_ − *C*_*g*2_	* − L_p_*
(Passive Pollution control, Passive Supervision, Participation)	*I*_*e*1_ * − pL_e_*	* −C*_*g*2_	*I*_*p*1_ − *C_p_ − L_p_*
(Passive Pollution control, Passive Supervision, Non-Participation)	*I*_*e*1_	*C*_*g*2_	−*L_p_*
(Positive pollution control, Positive Supervision, Participation)	*I*_*e*2_ + *I*_*e*3_ − *C_e_ + S*	*I_g_* − *C*_*g*1_ − *R − S*	*I*_*p*1_ + *I*_*p*2_ − *C_p_ + R*
(Positive pollution control, Positive Supervision, Non-Participation)	*I*_*e*1_ * − C_e_ + S*	*I_g_* − *C*_*g*1_ − *S*	*I*_*p*2_
(Positive pollution control, Passive Supervision, Participation)	*I*_*e*2_ * − C_e_ − I*_*e*3_	*I_g_*	*I*_*p*1_ + *I*_*p*2_ − *C_p_*
(Positive pollution control, Passive Supervision, Non-Participation)	*I*_*e*2_ * − C_e_*	*I_g_*	*I*_*p*2_

**Table 3:** Equilibrium points.

Participants	Government	
**Enterprise**	(1,1,0)	(0,1,1)
	(1,1,1)	(0,1,0)
**Public**	(1,0,0)	(0,0,1)
	(1,0,1)	(0,0,0)

**Table 4:** Variables and description.

Variable Type	Notation	Meaning	Unit	Description
Explained Variable	*Iwg*	Industrial waste gas	Million cu. m	Selecting the three industrial wastes as environmental control indicators [34].
	*Iww*	Industrial waste water	10,000 tons	
	*Isw*	Industrial solid waste	10,000 tons	
Explanatory variable	*Gwg*	Investment in industrial waste gas treatment	10,000 yuan/million cu.m	Government supervision
	*Gww*	Investment in industrial waste water treatment	10,000 yuan/Ten thousand tons	
	*Gsw*	Investment in industrial solid waste treatment	10,000 yuan/ 10,000 tons	
	*Pwg*	Number of public participation in industrial waste gas problem	10,000 times	Public participation in environmental governance through letters and visits, phone calls, and Internet
	*Pww*	Number of public participation in industrial waste water problem	10,000 times	
	*Psw*	Number of public participation in industrial solid waste problem	10,000 times	
Control variable	*Psg*	The proportion of secondary industry to GDP	%	Regional industrial layout is closely related to environmental quality. The larger the proportion of secondary industry, the worse the environmental quality [35].
	*Neu*	Number of enterprises per unit area	Number/square kilometer	The greater the density of enterprises, the more serious the environmental problem [36].
	*Dpe*	degree of public education (Average Years of Education)	Year	The more educated the public is, the more attention they will pay to environmental issues, which in turn will improve environmental conditions [37].

**Table 5:** The results of spatial error model and spatial lag model.

Variable	Spatial Error Model			Spatial Lag Model		
	*Iwg*	*Iww*	*Isw*	*Iwg*	*Iww*	*Isw*
*Gwg*	−0.0365 * (−1.9778)			−0.0411 ** (−2.4748)		
*Gww*		−0.6824 * (−1.8778)			−0.7647 * (−2.1381)	
*Gsw*			−0.0068 (−1.046)			− 0.0041 * (−2.1283)
*Pwg*	−0.0111 * (−1.8061)			−0.0066 **		
*Pww*		−0.0013 * (−1.9614)			−0.0026 * (−2.0116)	
*Psw*			−0.0069 * (−2.2086)			−0.0076 * (−1.9754)
Gwg*Pwg	−0.087 ** (−2.1017)			−0.1736 ** (−2.3383)		
Gww*Pww		−0.1146 * (−2.0747)			−0.1654 * (−2.0353)	
Gsw*Psw			−0.1416 * (−2.3028)			−0.0786 ** (−2.1017)
*Psg*	0.1143 (1.2355)	0.1641 (1.5002)	0.0662 (1.5410)	0.0886 (1.0274)	0.1753 * (1.9651)	0.0712 * (2.2356)
*Neu*	0.0602 (1.1122)	0.0501 * (1.7254)	0.0602 (1.0701)	0.0563 (1.6107)	0.0441 * (2.2417)	0.0613 (1.2221)
*Dpe*	−0.0029 (−1.3168)	−0.0008 * (−1.5062)	−0.0023 (−1.0059)	−0.0024 ** (−2.0846)	−0.0016 * (−1.5313)	−0.0026 (−1.3059)
*C*	−46.2304 (−1.2241)	−18.9251 (−0.9884)	210.8751 (1.1452)	−44.9762 (−1.2247)	−11.7671 * (−2.2085)	215.7221 (0.9061)
*Adj R* ^2^	0.6861	0.7001	0.5872	0.9503	0.8762	0.7681
*F* value	18.8531	22.9802	37.9052	33.7651	29.9765	45.8711

Note: The t-test value is in brackets. *, ** are significant at the levels of 10%, 5%, and 1% respectively.
